# Podcasting in Medicine: A Review of the Current Content by Specialty

**DOI:** 10.7759/cureus.6726

**Published:** 2020-01-21

**Authors:** Andrew Little, Zach Hampton, Tanner Gronowski, Cameron Meyer, Andrew Kalnow

**Affiliations:** 1 Emergency Medicine, OhioHealth Doctors Hospital, Columbus, USA; 2 Emergency Medicine, Academic Life in Emergency Medicine (ALiEM), Boise, USA

**Keywords:** podcasts, podcasting

## Abstract

Background

Podcasts and their use in medical education is growing and becoming more popular, all while not knowing what podcasts are available for each specialty.

Objectives

To ascertain the number of podcasts available by specialty and collect basic characteristics of each podcast.

Methods

This was a Google-based, investigational study of medical podcasts by specialty undertaken by all authors from January to June 2019. Search terms included “podcasts in ____”, where various specialties were inserted to identify current podcasts.

Results

Over the course of a six month period, 19 specialties were investigated for podcasting content. Emergency medicine, internal medicine, and pediatrics had the most active podcasts. Obstetrics and gynecology, ophthalmology, and orthopedic surgery have the most inactive podcasts. Neurosurgery was the only specialty searched without any identifiable active podcasts.

Conclusions

While emergency medicine has a large number of podcasts, both active and available other specialties have less of a selection.

## Introduction

Medical podcasts have greatly increased in number and popularity over the past 20 years [1]. From medical school lectures to continued medical education, podcasts are increasingly used for dissemination of medical knowledge [2-4]. Podcasts are digital audio files made available on the Internet for downloading to a computer or mobile device, typically available as a series, new installments of which can be received by subscribers automatically [5]. For decades, podcasts have been used not only for personal enjoyment but also for dissemination of knowledge [6-7]. The 2018 Infinite Dial report, a longstanding series of reports that covers consumer usage of media and technology, found that an estimated 124 million people currently listen to podcasts [8]. Of these, approximately 73 million have listened in the last month. On average, this large survey found that the average listener consumes seven podcasts per week [8]. Separate articles note that there are more than 500,000 active podcasts in 100 languages on Apple podcast alone [9-10]. Given the large number of listeners that can be reached via this platform, it is not surprising that medically oriented podcasts exist for circulation of medical knowledge for all levels of learning, from medical student to attending, and podcasts as a platform for medical education have grown significantly over the past decade [6].

There is little evidence to suggest standards for creating podcasts, though several non-evidence-based resources exist [11-12]. Additionally, there is a lack of research suggesting optimal lengths, platforms, and delivery methods. Regardless, despite the increase in popularity of this medium, there has yet to be an investigation into the podcasts currently available for learner consumption. To this point, there has been no systematic review of these resources by specialty. Several studies have demonstrated the ability of podcasts as an educational tool to increase learner retention (Geyer H, Beylefeld A, Hugo A: To podcast or not to podcast? Students’ feedback on a different learning experience in histology. Presented at the European Conference on E-Learning, Ayia Napa, Cyprus, November 7, 2008) [13-16]. Despite the increase in the number of podcasts available for those in the medical community, many have succumbed to what has been termed “pod fade,” a phenomenon where podcasters stop producing content, which usually occurs over six months [17]. Although this is a well-documented phenomenon in the non-medical podcast community, it has never been evaluated in the medical podcasting arena [17]. Understandably, finding available podcasts currently producing new material can be challenging and often frustrating, especially to the learner.

This study represents a Google-based review of current podcasts by specialty, with the specific aim of better defining current availability, content, and characteristics surrounding these podcasts.

## Materials and methods

This was a Google-based, investigational study of medical podcasts by specialty undertaken by all authors from January to June 2019. Search terms included “podcasts in ____”, where various specialties were inserted to identify current podcasts. After performing this search for each specialty, we set out to find 50 podcasts per specialty. The search was conducted using the Google search engine and was carried through the first five search pages. Fifty sites were reviewed. The first step during the review was determining whether the site represented an actual podcast. Exclusion criteria included third party podcasting websites and websites where the information in question was not available. Also, individual episodes about a subject, but not part of a “medical specialty podcast,” were not included in our data. All other podcasts were included. Once a podcast was identified, the first determination was "active" versus "inactive". For this definition, we deemed any podcast that had not released an episode within six months prior to the search as being "inactive". For each podcast, additional data was collected, including the number of available episodes, podcast name, release schedule, average run time, and current host(s).

Podcasts were searched by specialty, including anesthesiology, dermatology, emergency medicine, family medicine, internal medicine, general surgery, neurology, neurosurgery, obstetrics and gynecology, ophthalmology, orthopedic surgery, otolaryngology, pathology, pediatrics, physical medicine and rehabilitation, plastic surgery, psychiatry, radiology, and urology. 

Rarely, some information was not readily available for review. We used a "best-effort" approach to obtain as much information from the available podcasts. When necessary, information was also obtained from podcast hosting platforms, such as iTunes (Apple, Inc., Cupertino, CA), if not readily available on the website. When information could not be obtained, the term “indeterminate” was used. 

We performed other analysis including episodes available per specialty, the average length of episodes per specialty, and total hours of content available per specialty. To describe the release schedule, the following terms were used: weekly, bimonthly, monthly, series, sporadic, and intermittent. For this study, we used the term "sporadic" to define podcasts without a clear regularity to their release schedule. Average podcast runtime was estimated by calculating the mean runtime of the most recent five episodes. 

## Results

Overall, 19 specialties were investigated for podcasting content. Table 1 shows the results for those podcasts deemed "active" or "inactive." Emergency medicine (28), internal medicine (13), and pediatrics (13) were found to have the most active podcasts (active podcasts number in parentheses). Obstetrics and gynecology (5), ophthalmology (5), and orthopedic surgery (5) were found to have the most inactive podcasts. Neurosurgery was the only specialty searched without any identifiable active podcasts (had only one inactive podcast). We performed other analyses, including episodes available per specialty (Table 2), the average length of episodes per specialty (Table 3), and total hours of content available per specialty (Table 4).

**Table 1:** Active, Inactive, and Total Podcasts by Specialty

Specialty	Inactive podcasts	Active Podcasts	Total Podcasts
Anesthesiology	4	8	12
Dermatology	1	9	10
Emergency Medicine	4	28	32
Family Medicine	3	10	13
Internal Medicine	2	13	15
General Surgery	2	10	12
Neurology	0	6	6
Neurosurgery	3	0	3
Obstetrics and Gynecology	5	10	15
Ophthalmology	5	5	10
Orthopedic Surgery	5	5	10
Otolaryngology	4	6	10
Pathology	2	2	4
Pediatrics	0	13	13
Physical Medicine and Rehabilitation	1	3	4
Plastic Surgery	2	7	9
Psychiatry	3	9	12
Radiology	1	7	8
Urology	1	1	2

**Table 2:** Total Episodes Per Specialty in Descending Order

Specialty	Total episodes
Emergency Medicine	2,434
Internal Medicine	1,374
Family Medicine	1,031
General Surgery	1,004
Pediatrics	964
Ophthalmology	941
Obstetrics and Gynecology	920
Neurology	864
Anesthesia	726
Dermatology	699
Psychiatry	629
Radiology	527
Orthopedic Surgery	411
Otolaryngology	403
Plastic Surgery	400
Pathology	212
Urology	136
Neurosurgery	94
Physical Medicine and Rehabilitation	70

**Table 3:** Average Minutes Per Episode by Specialty in Descending Order

Specialty	Average minutes per episode
Emergency Medicine	36.6
Pediatrics	35.2
Family Medicine	33
Dermatology	32
Physical Medicine and Rehabilitation	30.5
Neurosurgery	30
Plastic Surgery	29.7
Pathology	29.2
Ophthalmology	28
Anesthesia	27.4
Psychiatry	27.4
Radiology	26.4
Internal Medicine	25.3
Obstetrics and Gynecology	24.9
Otolaryngology	23.7
General Surgery	22.2
Neurology	17.5
Urology	17
Orthopedic Surgery	15.4

**Table 4:** Estimated Hours of Content by Specialty in Descending Order. Determined by Number of Episodes Multiplied by Average Minutes Per Episode Divided by 60 Minutes

Specialty	Estimated hours of content
Emergency Medicine	1,485
Internal Medicine	579
Family Medicine	567
Pediatrics	566
Ophthalmology	439
Obstetrics and Gynecology	382
Dermatology	373
General Surgery	372
Anesthesia	332
Psychiatry	287
Neurology	252
Radiology	232
Plastic Surgery	198
Otolaryngology	159
Orthopedic Surgery	106
Pathology	103
Neurosurgery	47
Urology	39
Physical Medicine and Rehabilitation	36

Individual podcast information was summarized and can be found below. 

Anesthesiology

For anesthesiology, there was a total of 12 podcasts found, eight of which were active. There was a total of 726 episodes with one weekly podcast providing 41% of the episodes. After removing that outlier from the data, the remainder of the podcasts still averaged almost 39 episodes per podcast. As far as the frequency of content being published, two podcasts put out episodes weekly. However, the overwhelming majority provided content sporadically which was found to be similar to other specialties. They had the 9th-most active podcasts, 9th-most total episodes, and 9th-most estimated hours of content. The average podcast episode in anesthesia was 27.4 minutes per episode. 

Dermatology

For dermatology, there was a total of 10 podcasts found, nine of which were active. There was a total of 699 episodes with one podcast providing 43% of the episodes. After removing that outlier from the data, the remainder of the podcasts still averaged 45 episodes per podcast. As far as the frequency of content being published, one podcast is published weekly, four monthly, and the remainder were sporadic. They were tied for the 7th-most active podcasts. They had the 10th-most total episodes and the 7th-most estimated hours of content. The average podcast episode was 32 minutes per episode.

Emergency medicine

For emergency medicine, there was a total of 32 podcasts found, 28 of which were active. There was a total of 2,434 episodes. No one podcast made up more than 1% of the total content. The average podcast contained 76 episodes. Four podcasts put out episodes weekly, one bi-monthly, 13 monthly, and 14 were sporadic. They had the most active podcasts, the most total episodes, and the most estimated hours of content. The average podcast episode in emergency medicine was 36.6 minutes per episode. By far, emergency medicine put out the most content of any specialty and the content was the most evenly produced by all of its podcasts. 

Family medicine

For family medicine, there was a total of 13 podcasts found, 10 of which were active. There was a total of 1,031 episodes with one podcast providing almost 36% of the episodes. After removing that outlier from the data, the remainder of the podcasts still averaged 56 episodes per podcast. Two podcasts put out episodes weekly, two bi-monthly, two monthly, and seven were sporadic. They were tied for the 4th-most active podcasts. They had the 3rd-most total episodes and the 3rd-most estimated hours of content. The average podcast episode was 33 minutes per episode.

Internal medicine

For internal medicine, there was a total of 15 podcasts found, 13 of which were active. There was a total of 1,374 episodes with two podcasts having over 200 episodes. Altogether, the podcasts averaged 92 episodes per podcast. Two podcasts put out episodes weekly, three bi-monthly, four monthly, and six were sporadic. They were tied for the 2nd-most active podcasts. They had the 2nd-most total episodes and 2nd-most estimated hours of content. The average podcast episode was 25.3 minutes per episode.

General surgery

For general surgery, there was a total of 12 podcasts found, 10 of which were active. There was a total of 1,004 episodes with two podcasts providing almost 48% of the episodes. After removing those podcasts from the data, the remainder of the podcasts averaged 53 episodes per podcast. Two podcasts put out episodes weekly, two monthly, and eight were sporadic. They were tied for the 4th-most active podcasts, 4th-most total episodes, and the 8th-most estimated hours of content. The average podcast episode was 22.1 minutes per episode.

Neurology

For neurology, there was a total of six podcasts found, all of which were active. There was a total of 864 episodes with two podcasts providing over 69% of the episodes. Altogether, the podcasts averaged 92 episodes per podcast. Three podcasts put out episodes weekly, one bi-monthly, zero monthly, and two were sporadic. They were tied for the 12th-most active podcasts. They had the 8th-most total episodes and the 11th-most estimated hours of content. The average podcast episode was 17.5 minutes per episode.

Neurosurgery

For neurosurgery, there was a total of three podcasts found, none of which were active. There was a total of 94 episodes with one podcast providing over 84% of the episodes. The other two podcasts averaged 7.5 episodes. All were published sporadically. They had the least active podcasts, the second to the last most total episodes and third to last most estimated hours of content. The average podcast episode was 24.9 minutes per episode.

Obstetrics and gynecology 

For obstetrics and gynecology, there was a total of 15 podcasts found, 10 of which were active. There was a total of 920 episodes with one podcast providing over 32% of the episodes. Excluding that outlier, altogether the podcasts averaged 45 episodes per podcast. Three podcasts put out episodes weekly, two bi-monthly, two monthly, and eight were sporadic. They were tied for the fourth-most active podcasts. They had the seventh-most total episodes and the sixth-most estimated hours of content. The average podcast episode was 24.9 minutes per episode.

Ophthalmology

For ophthalmology, there was a total of 10 podcasts found, five of which were active. There was a total of 941 episodes with three podcasts providing all but 15% of the total episodes. Two podcasts put out episodes weekly, one bi-monthly, zero monthly, and seven were sporadic. They were tied for the 14th-most active podcasts. They had the sixth-most total episodes and the fifth-most estimated hours of content. The average podcast episode was 28 minutes per episode. 

Orthopedic surgery

For orthopedic surgery, there was a total of 10 podcasts found, five of which were active. There was a total of 411 episodes with one podcast providing over 52% of the total episodes. After removing that outlier the average podcast averaged 22 episodes. There was one podcast that put out episodes weekly, and the rest were sporadic. They were tied for the 14th-most active podcasts. They had the 13th-most total episodes and the 15th-most estimated hours of content. The average podcast episode was the shortest at 15.4 minutes per episode. 

Otolaryngology

For otolaryngology, there was a total of 10 podcasts found, six of which were active. There was a total of 406 episodes with over 61% of the episodes coming from two podcasts. There was one podcast that put out episodes biweekly, two monthly, and three were sporadical. They were tied for the 12th-most active podcasts. They had the 14th-most total episodes and the 14th-most estimated hours of content. The average podcast episode was 23.7 minutes per episode.

Pathology

For pathology, there was a total of four podcasts found, two of which were active. There was a total of 212 episodes with the average podcast having 53 episodes. All the podcasts were published sporadically. They had the seventh-most active podcasts, the 16th-most total episodes, and the 16th-most estimated hours of content. The average podcast episode was 29.2 minutes per episode.

Pediatrics

For pediatrics, there were a total of 13 podcasts found and all were active. There was a total of 964 episodes with one podcast providing 24% of the total episodes. Excluding that outlier, the average episodes per podcast were 61. There was one podcast that put out episodes weekly, three monthly, and eight were sporadic. They were tied for the second-most active podcasts. They had the fifth-most total episodes and the fourth-most estimated hours of content. The average podcast episode was 35.2 minutes per episode.

Physical medicine and rehabilitation

For physical medicine and rehabilitation, there was a total of four podcasts found and three were active. There was a total of 70 episodes with two podcasts providing 79% of the total episodes. Two podcasts put out episodes monthly and two were published sporadically. They had the 16th-most active podcasts, the fewest total episodes, and the fewest estimated hours of content. The average podcast episode was 30.5 minutes per episode.

Plastic surgery 

For plastic surgery, there was a total of nine podcasts found and seven were active. There was a total of 400 episodes and the average episodes per podcast were 44.4. Four podcasts put out episodes weekly, three monthly, and two were sporadic. They were tied for the 10th-most active podcasts. They had the 15th-most total episodes and the 13th-most estimated hours of content. The average podcast episode was 29.7 minutes per episode.

Psychiatry 

For psychiatry, there was a total of 12 podcasts found, nine of which were active. There was a total of 629 episodes with one podcast providing over 23% of the episodes. Excluding that outlier, altogether the podcasts averaged 40 episodes per podcast. One podcast put out episodes weekly, two bi-monthly, two monthly, and seven were sporadic. They were tied for the seventh-most active podcasts. They had the 11th-most total episodes and the 10th-most estimated hours of content. The average podcast episode was 27.4 minutes per episode.

Radiology

For radiology, there was a total of eight podcasts found, seven of which were active. There was a total of 527 episodes with one podcast providing almost 49% of the episodes. Excluding that outlier, altogether the podcasts averaged 30 episodes per podcast. One podcast published bimonthly, one monthly, and six were sporadic. They were tied for the 10th-most active podcasts. They had the 12th-most total episodes and the 12th-most estimated hours of content. The average podcast episode was 26.4 minutes per episode.

Urology

For urology, there were a total of two podcasts found, one of which was active. There was a total of 136 episodes with the average episodes per podcast being 68 with one podcast that put out episodes weekly and one that published sporadically. They had the second least active podcasts, the third to the last most total episodes, and the second to the last most estimated hours of content. The average podcast episode was 17 minutes per episode.

## Discussion

This study identifies wide variability in the creation of podcasts in medicine by the specialties searched, with some specialties producing a large volume of podcast episodes monthly and other specialties not actively producing any. While it has been documented that podcasts in medicine have greatly increased in number and popularity over the past 20 years, this has not been evenly applicable across specialties. Emergency medicine was not only found to have the most active podcasts but the highest volume of content available, more than twice that found when combining internal medicine with obstetrics and gynecology, respectively (the next two most active podcasting specialties). A few others have either limited or no active content available for their learners, leaving open potential new content creation for members of these specialties (neurosurgery, pathology, physical medicine and rehabilitation, and urology). Given the increasing popularity of podcasting in medicine, including learner favorability in listening to podcasts as a way to receive information, there is significant room in most specialties to expand content with specialty-specific podcasts. The authors feel this study represents a first attempt to compile a working list of podcasts by specialty, searched for in a way similar to how a learner would attempt to access this information, creating the foundation for further investigation into the utilization of specialty-specific podcasts in medicine. 

## Conclusions

Although a few specialties have provided large amounts of content to their learners, others are lacking in both the number of podcasts available and specifically in the number of active podcasts. Although this study cannot make conclusions about the quality of these podcasts or learner utilization, the aim is to provide the first categorically indexed review of available podcasts within the search parameters outlined above. Learners who use medical podcasts will ideally find this article helpful in identifying content. These investigators hope that physicians in underrepresented specialties will see the value in providing this content and will take the initiative to create and disseminate new podcasts. 
